# The complete mitochondrial genome of the hybrid offspring *Epinephelus fuscoguttatus ♀* × *Epinephelus tukula ♂*

**DOI:** 10.1080/23802359.2019.1644554

**Published:** 2019-07-23

**Authors:** Meiling Cheng, Yongsheng Tian, Zhentong Li, Linna Wang, Yuping Wu, Jingjing Zhang, Zunfang Pang, Wenhui Ma, Jieming Zhai

**Affiliations:** a Key Laboratory of Sustainable Development of Marine Fisheries, Ministry of Agriculture and Rural Affairs, Yellow Sea Fisheries Research Institute, Chinese Academy of Fishery Sciences, Qingdao, China;; b College of Fisheries and Life Science, Dalian Ocean University, Dalian, China;; c Laboratory for Marine Fisheries Science and Food Production Processes, Qingdao National Laboratory for Marine Science and Technology, Qingdao, China;; d College of Fisheries and Life Science, Shanghai Ocean University, Shanghai, China;; e Laizhou Mingbo Aquatic Co., Ltd, Yantai, China

**Keywords:** Mitochondrial genome, *Epinephelus fuscoguttatus ♀ × Epinephelus tukula ♂*

## Abstract

The hybrid offspring *Epinephelus fuscoguttatus* ♀ × *E. tukula ♂* showed heterosis in terms of growth and disease resistance. The mitochondrial genome is 16,629 bp in length and consists of 13 protein-coding genes, 2 rRNA genes, 22 tRNA genes, and a control region. The difference of total length is mainly caused by the difference of length in non-coding region. The nucleotide base composition is A = 29.15%, G = 15.62%, T = 26.91%, C = 28.32%, A + T = 56.06%, and C + G = 43.94%. The phylogenetic analysis using neighbour-joining (NJ) method showed that the hybrid offspring has a closer relationship to *E. fuscoguttatus* × *E. lanceolatus.*

*Epinephelus fuscoguttatus* and *E. tukula* both belonged to the subfamily *Epinephelinae*. The hybrid offspring was obtained by *E. fuscoguttatus* ♀ × *E. tukula* ♂ using artificial insemination. The hybrid offspring showed heterosis in terms of growth and disease resistance. In this study, PCR amplification and primer walking sequence method were used to analysis the complete mitogenome of *E. fuscoguttatus* ♀ × *E. tukula* ♂, and the sequencing results were spliced, annotated, and analyzed by bioinformatics software. Analysis of its genetic background is conducive to breeding and genetic improvement of varieties.

Hybrid *E*. *fuscoguttatus* ♀ × *E. tukula* ♂ specimens were sampled from Laizhou Mingbo Aquatic Co., Ltd., Shandong province, China (372°5′6.73″N 120°0′15.11″E). Samples stored in a refrigerator of −80 °C with accession number 20181204EFET01. The complete mitochondrial genome of hybrid offspring is 16,629 bp in length (Gen Bank, MK408449), which has the structural characteristics of mitochondrial genes and is mainly composed of non-coding region and coding region. The non-coding region is divided into two segments: light chain replication initiation region and control region. The coding region consists of 13 protein-coding genes, two rRNA genes, and 22 tRNA genes. In addition, only ND6 and eight tRNA genes were encoded in the light chain, and the remaining genes were all encoded in the heavy chain. There were nine gene intervals in the mitochondrial genome of this fish, and the longest distance between the tRNA-asn and tRNA-cys genes was 39 bp. The overall base composition were A = 29.15%, G = 15.62%, T = 26.91%, C = 28.32%, A + T = 56.06%, and C + G = 43.94%.

In order to verify the evolutionary relationship, we perform multiple sequence alignment and construct a neighbour-joining (NJ) phylogenetic tree (Figure 1) (Tamura et al. 2013). As shown, the hybrid offspring was closer with *E. fuscoguttatus* × *E. lanceolatus* (Genbank, KY132101) (Zhu K et al. 2016).

**Figure 1.**
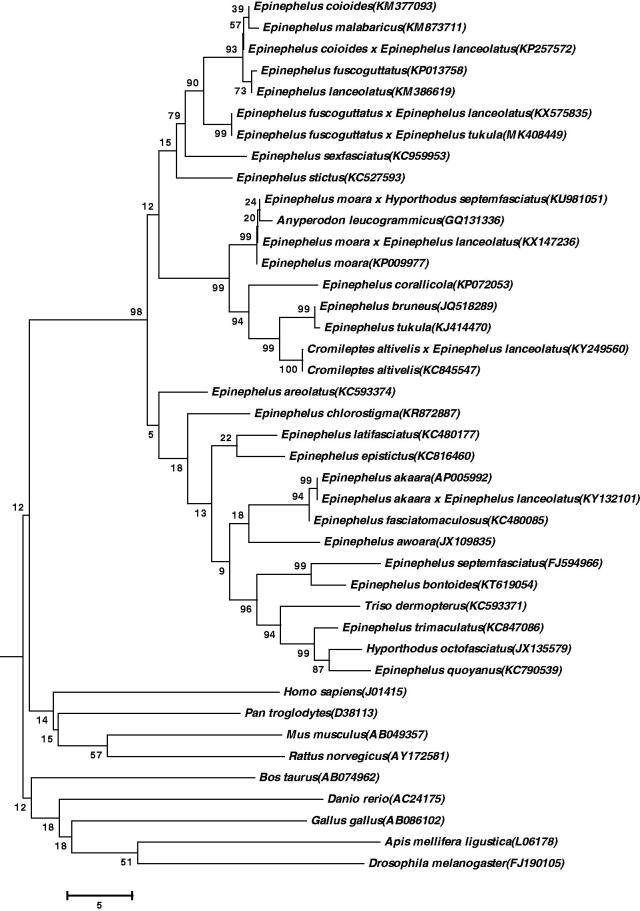
The phylogenetic tree of 41 species. Numbers on each node are bootstrap values of 1000 replicates.
